# Whole-Body Cryostimulation in Post-COVID Rehabilitation for Patients with Obesity: A Multidisciplinary Feasibility Study

**DOI:** 10.3390/biomedicines11113092

**Published:** 2023-11-18

**Authors:** Jacopo Maria Fontana, Angelo Alito, Paolo Piterà, Federica Verme, Stefania Cattaldo, Mauro Cornacchia, Stefania Mai, Amelia Brunani, Paolo Capodaglio

**Affiliations:** 1 Research Laboratory in Biomechanics, Rehabilitation and Ergonomics, IRCCS, Istituto Auxologico Italiano, San Giuseppe Hospital, 28824 Piancavallo, Italy; p.pitera@auxologico.it (P.P.); f.verme@auxologico.it (F.V.); brunani@auxologico.it (A.B.); p.capodaglio@auxologico.it (P.C.); 2 Department of Biomedical, Dental Sciences and Morphological and Functional Images, University of Messina, 98125 Messina, Italy; alitoa@unime.it; 3 Laboratory of Clinical Neurobiology, IRCCS, Istituto Auxologico Italiano, San Giuseppe Hospital, 28824 Piancavallo, Italy; s.cattaldo@auxologico.it; 4 Pulmonary Rehabilitation Department, IRCCS, Istituto Auxologico Italiano, San Giuseppe Hospital, 28824 Piancavallo, Italy; m.cornacchia@auxologico.it; 5 Laboratory of Metabolic Research, IRCCS, Istituto Auxologico Italiano, San Giuseppe Hospital, 28824 Piancavallo, Italy; s.mai@auxologico.it; 6 Department of Surgical Sciences, Physical and Rehabilitation Medicine, University of Torino, 10121 Torino, Italy

**Keywords:** multidisciplinary rehabilitation, obesity, personalized rehabilitation, post-COVID-19 condition, rehabilitation, whole-body cryostimulation

## Abstract

Background: A post-COVID condition can reduce activity and quality of life, resulting in a significant socioeconomic and health burden. Understanding its impact on patients’ health is important for the development of personalized rehabilitation interventions. An independent association between obesity and post-COVID condition was found because of complications and comorbidities. Methods: Sixteen patients with obesity and post-COVID symptoms (i.e., dyspnea, pain, poor sleep quality, muscle fatigue), admitted to the Istituto Auxologico Italiano, Piancavallo (VB), Italy, were recruited for a four-week rehabilitation program including conventional exercise therapy, nutritional intervention, psychological support and whole-body cryostimulation (WBC). Results: All participants attended all sessions of the program. Anthropometric data showed statistically significant changes in weight, waist circumference and body mass index. Biochemical analyses showed significant reductions in lipid and inflammatory profiles. There was a significant improvement in physical performance, reduction in pain and improvement in psychological well-being. Conclusion: A multidisciplinary rehabilitation protocol including WBC, designed for patients with obesity and a post-COVID condition, is safe and feasible. The overall improvements demonstrate that multidisciplinary rehabilitation was effective on post COVID patients and suggest that the use of WBC is safe and could play a role as a booster in rehabilitation programs.

## 1. Introduction

In December 2019, the world saw the emergence of Severe Acute Respiratory Syndrome Coronavirus 2 (SARS-CoV2), which can range from an asymptomatic infection to acute respiratory distress syndrome (ARDS) [1,2]. The new coronavirus disease 2019 (COVID-19) put health systems in serious difficulties, infecting over 635 million people with more than 6.6 million deaths [3,4]. Nowadays, as the pandemic persists, more than 30% of people infected with COVID-19, including asymptomatic cases, and approximately 80% of patients hospitalized for COVID-19 have reported long COVID, or post COVID-19 condition (PCC) after infection [5]. PCC is defined as sequelae of symptoms that persist for at least 4 weeks after acute infection or 3 months after infection, for at least 2 months and that cannot be explained by other causes [6]. This condition is characterized by dyspnea, coughing, fever, and persistent loss of smell or taste which eventually combines with musculoskeletal (e.g., fatigue and myalgias) and psychological (e.g., concentration and memory disorders, depression, and anxiety) problems [5,7,8]. PCC could reduce activity ability and health-related quality of life (HR-QoL), imposing significant socioeconomic and health burdens, and has attracted widespread attention [9]. Understanding PCC impact on patients’ health and well-being is of great importance in developing effective and personalized rehabilitation and pharmacological interventions [10,11]. 

Obesity is linked to the progression of COVID-19 through a number of molecular pathways that increase SARS-CoV-2 infection vulnerability [12]. Adipose tissues in obese patients have a greater number of proteases and receptors for SARS-CoV-2 entry, suggesting that they could act as an accelerator and reservoir for this virus, enhancing the immune response and systemic inflammation [12]. 

Several studies have documented an independent association between obesity and PCC because of the complications and comorbidities, reporting a 25% higher risk of PCC with an additional burden on the immune system and involvement of physical and physiological processes [13,14,15].

Other studies have reported that patients with prolonged COVID symptoms are more likely to have obesity and any obesity degree (BMI ≥ 30 kg/m^2^) was associated with a worse PCC prognosis [16]. Thus, an intensive multidisciplinary, tailored rehabilitation approach is required to maximize the patient’s functional recovery and facilitate the returning to pre-morbid life, especially when PCC symptoms appear to persist or even worsen in susceptible individuals [17]. 

Conventional rehabilitative approaches for PCC include physiotherapy [18,19], breathing [20] and resistance and/or aerobic exercises [21], psychological counseling [22], and home-based programs [23]. 

Physical therapy in PCC focuses on improving physical strength, endurance, balance, and mobility, while respiratory therapy aims to improve lung function and breathing patterns [18]. Psychological counseling is essential to address the mental and emotional impact of PCC, including anxiety, depression, and post-traumatic stress disorder [22]. However, it is important to explore alternative interventions that can complement and enhance the conventional rehabilitation process. 

Previous studies investigated Whole-Body Cryostimulation (WBC) as a treatment able to reduce pain and inflammatory status in several conditions [24] and to improve depression, anxiety [25], functional status and fatigue [26], and sleep quality [27]. WBC consists of exposure to cryogenic temperatures (−110 °C to −140 °C) for a short period of time (2–3 min) and is a therapy with widely reported anti-inflammatory and less studied metabolic effects [28]. It is used as a post-exercise recovery technique and as an adjuvant therapy in conditions of rehabilitation interest [29,30,31] such as rheumatoid arthritis, fibromyalgia, multiple sclerosis, sleep disturbances, obesity. Moreover, the positive effects of ten serial sessions of WBC have been previously reported in patients with PCC [32].

The effect of adding WBC to a multidisciplinary rehabilitation program has not been studied extensively, particularly in the context of post-COVID care, although it is a unique approach [32]. The purpose of this study was to investigate the safety, acceptability and feasibility of a multidisciplinary personalized rehabilitation program including WBC in patients with obesity and PCC, admitted to a rehabilitation unit, and to provide additional data on cryostimulation as an adjuvant treatment for functional recovery.

## 2. Materials and Methods

### 2.1. Study Design

A single arm longitudinal study was performed. According to the literature [33], feasibility studies are an attempt to answer questions about whether some aspect of a future trial is feasible, in this case the authors seek to determine the acceptability of an intervention and the perceived importance of types of outcomes. Acceptability can be interpreted as the participants’ positive or negative opinion of a particular innovation. Patients were recruited from the Rehabilitation and Pneumology Unit of the San Giuseppe Hospital, Istituto Auxologico Italiano, Piancavallo (VB), Italy. The participants engaged in a 4-week multidisciplinary rehabilitation intervention. This study was conducted as part of a line of research aimed at defining better personalized rehabilitation programs for patients with obesity. 

### 2.2. Participant Eligibility

Inclusion criteria were age 18–75 years, BMI ≥ 30 kg/m^2^, and PCC. Exclusion criteria were severe psychiatric illness, acute respiratory disease, acute cardiovascular disease, unstable hypertension, cold intolerance, claustrophobia, pregnancy, recent change in usual medication, previous treatment with WBC, weight loss in the previous 3 months, and body temperature > 37.5 °C.

### 2.3. Participants

Between July 2021 and September 2022, 16 patients admitted to the Rehabilitation and Pneumology Unit of the San Giuseppe Hospital, Istituto Auxologico Italiano, Piancavallo (VB), Italy, agreed to participate in the study.

### 2.4. Study Variables

Anthropometric data, cardiovascular parameters, blood tests and functional test scores were collected at baseline (T0) and within 4 weeks at the end of the rehabilitation protocol (T10). 

**Figure 1:**
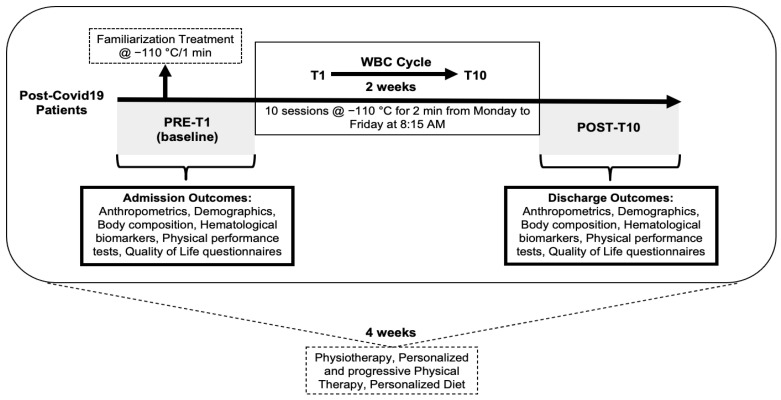
Schematic of the study design showing the timeline of the study protocol and research outcomes.

A schematic diagram of the protocol can be found in Figure 1. There were no follow-up measurements after discharge. Anthropometric measurements including weight, height, body mass index (BMI) and waist circumference (WC) were taken using a scale and tape measure. Resting cardiovascular parameters, including heart rate (HR) and systolic and diastolic blood pressure (SBP/DBP), were measured by a trained health professional using standard procedures. Hematological biomarkers analyzed from morning fasting blood samples included glucose, total cholesterol, high-density lipoprotein (HDL), low-density lipoprotein (LDL), triglycerides (TG) and C-reactive protein (CRP) measured by standard laboratory techniques, specifically enzymatic methods for glucose, cholesterol, and triglycerides, immunoturbidimetric assay for CRP and spectrophotometric methods for HDL and LDL.

Physical performance tests included: a 6-min walk test (6MWT) [34] and the Timed Up and Go test (TUG) [35]. General pain was assessed using the Visual Analogue Scale (VAS) [36].

The Psychological General Well-Being Index (PGWBI) was used to measure subjective psychological well-being [37].

Patients admitted to the Pneumology Department had their basal SpO2 parameters measured by polysomnography on admission and discharge.

### 2.5. Intervention 

The multidisciplinary rehabilitation program included individualized nutritional intervention, psychological support and supervised physical activity throughout the hospital stay. All patients received a balanced, hypocaloric Mediterranean diet consisting of three meals a day with 18–20% protein, 27–30% fat (of which <8% saturated fat) and 50–55% carbohydrates (<15% simple sugars), and 30 g of fiber from fresh vegetables. Under the supervision of a physiotherapist, two 60-min physiotherapy sessions were performed daily, consisting of personalized progressive aerobic training, postural control exercises and progressive strengthening exercises. The aerobic sessions were monitored with subjective perception of fatigue (Borg’s CR10 scale) and oxygen saturation (SpO2). Exercise was stopped when a score of 5 was reached on the Borg scale. The first aerobic session, performed in the morning after WBC, consisted of walking at a predetermined cadence. The second session, performed in the afternoon, consisted of arm cranking at an intensity of 65% of HRmax according to Karvonen’s equation ((220 − age) × 0.65). This approach was individualized according to the patient’s fitness, clinical status, and subjective perception of fatigue. All patients underwent ten sessions of WBC over a 2-week period (1 treatment per day, Monday to Friday, at 8:15 am, before exercise classes and physiotherapy).

### 2.6. Description of the WBC Session

Subjects were exposed to extremely cold, dry air at −110 °C for 2 min in a cryochamber (Arctic, CryoScience, Rome, Italy). On the day before the first WBC session, a 1-min test session was performed. Sessions were conducted under the supervision of appropriately trained operators. On entering the cryochamber, the patients were asked to remove their glasses, contact lenses and jewelry, and to dry their bodies thoroughly to reduce the sensation of cold and avoid skin burns. Men were allowed to wear shorts or tracksuit bottoms (due to the severe cold sensation in some cases), a light t-shirt (or no shirt), mid-calf socks, clogs, gloves, headgear, and ear protection. Women also wore a sports bra or a light t-shirt. A surgical mask covered the nose and mouth. Subjects were encouraged to shift their weight, move their fingers, and breathe normally in the cryochamber. Visual and vocal contact with the volunteers was constant. For safety reasons, SBP and DBP blood pressure were measured before and after each treatment.

### 2.7. Feasibility

Adherence to the 4-week protocol treatment was monitored, and completion rates of tests and questionnaires before and after the intervention were assessed. Adverse events were monitored throughout the study. At the end of the intervention, an exit interview was conducted to collect qualitative information about the participants’ experience of the feasibility and effects of the intervention.

### 2.8. Statistical Analysis

Statistical analysis was performed using Jamovi statistical software version 2.4.8. Data were expressed as mean (±standard deviation). The Shapiro–Wilk test was used to evaluate the normality of the distribution of the data. Student’s paired *t* test for normal data and Wilcoxon’s nonparametric paired test for nonnormal data were used to compare admission and discharge data. The level of significance was set at *p* < 0.05. 

### 2.9. Ethical Considerations

Patients were fully informed of the scope and methodology of the study, which was conducted in accordance with the World Medical Association’s Declaration of Helsinki and approved by the Ethics Committee of Istituto Auxologico Italiano (reference: 2021_05_18_14). Written and verbal informed consent was obtained from all experimental patients.

## 3. Results

### 3.1. Participant Flow

From July 2021 to September 2022, a total of 16 patients (three males, mean age 55.9 ± 7.51 years) met the eligibility criteria, were enrolled in this study, and started treatment. Eleven patients were recruited from the rehabilitation unit and five from the Pneumology unit. All patients completed the study protocol. Anthropometric, hematological, and functional data at baseline (T0) are shown in Table 1.

#### 3.1.1. Admission and Discharge Data Comparison

After the four-week rehabilitation treatment, which included nutritional intervention, psychological support, supervised physical activity and WBC, the authors found the following changes. Anthropometric data showed statistically significant changes in weight, WC, and BMI, which were lower at discharge. Biochemical analyses showed a significant decrease in CRP, HDL, LDL, Tot Col, GLU at T10. Among the other parameters analyzed, no significant difference was found for TG at discharge. In terms of cardiovascular parameters, there was a significant reduction in SBP and HR but not in DBP. There was a significant improvement in performance capacity as measured by TUG and distance walked on the 6MWT, a significant reduction in pain as shown by VAS pain, and an improvement in psychological well-being as measured by the PGWBI scale. 

The patients hospitalized at the Pneumology Department showed significant improvements of Sp02 basal evaluation. A comprehensive overview of the results is provided in Table 2.

#### 3.1.2. Feasibility

All 16 participants attended all four weekly sessions of the program, indicating excellent compliance. The data collection completion rate was good overall. No adverse events occurred during the intervention period. Participants reported positive physical and mental changes and were generally satisfied with the program setting. Motivation was very high due to the innovative nature of the approach included in the protocol.

## 4. Discussion

The present study demonstrates the safety and the feasibility of a multidisciplinary rehabilitation program combined with WBC and provides some preliminary evidence in patients with obesity and PCC. 

In general, cold exposure has been shown to have systemic effects on the neuromuscular, autonomic, endocrine, cardiovascular and immune systems [24]. As obesity is characterized by chronic low-grade inflammation, the effect of WBC may be due to its anti-inflammatory properties, such as the reduction of pro-inflammatory responses such as reduced levels of inflammatory markers (e.g., CRP), modulation of the pro-oxidant/antioxidant balance, and the cytokine levels observed (i.e., TNF-α, IL-6, and IL-1) may help counteract the inflammatory processes associated with the PCC [38]. In addition, the biological effects of WBC are thought to be enhanced recovery through increased activation of the parasympathetic system and improved oxygenation of muscle tissue [39].

The correct diagnosis and management of PCC is challenging for healthcare providers due to the heterogeneity and complexity of clinical manifestations and the likely need for multidisciplinary management approaches [40,41,42]. 

The importance of identifying outcome measures in PCC rehabilitation, such as physical function, quality of life, general symptoms, disability, activities of daily living and return to work, is crucial [43]. Global assessment before and after a rehabilitation program should be undertaken to provide more evidence for the development of effective management plans for Long COVID patients [44], particularly in people at higher risk of developing more severe PCC, such as those with obesity [45].

In primary care, general practitioner prescription of physical activity in a safe environment with a trained facilitator, could be an extremely effective way to manage the symptoms of long COVID from the outset [46,47]. This prescription should be preceded by an initial consultation to identify comorbidities and risks such as post-exertional symptom exacerbation, which is characterized by a worsening of symptoms after physical or mental exertion, usually 12–48 h after the activity and lasting for several days or (rarely) weeks [48]. 

Exercise-based rehabilitation is a therapeutic approach that may play an important role in improving sympathovagal balance and normalizing sympathetic index levels [49].

Several studies have focused on developing the most appropriate exercise protocol for patients with PCC, ranging from strength and endurance exercises to combined aerobic and resistance training [22,50,51], which can be varied in intensity and duration, and all show improvements in functional capacity [17,52]. 

According to our results, the effects of exercise therapy on SpO2 levels and cardiovascular fitness in patients with obesity and PCC have been widely documented [53]. 

Research has demonstrated the beneficial effects of exercise in reducing inflammation, improving immune function, and promoting overall physical and mental well-being in people with a post-COVID condition [54,55,56]. 

Given the existing evidence of clinical and functional benefits following WBC in musculoskeletal, neurological, and psychological conditions, the addition of such treatment aims to improve the patient’s overall physical performance and perceived quality of life [57]. 

Indeed, WBC is a safe and innovative method capable of applying precise and homogeneous “doses” of cold and inducing a rapid systematic reduction of inflammation and oxidative stress [58] with therapeutic effects on fatigue, pain, thymic tone, depression and sleep [24,25], as well as metabolic effects [29] such as increased thermogenesis and improved lipid profile, insulin sensitivity and glucose utilization, and could thus enhance the beneficial effects of an exercise program, especially one of short duration [59,60,61].

Thus, in line with our previous studies, the benefits of WBC appear to be rapid, from the very first sessions, probably due to its rapid anti-inflammatory effect [62]. So, implementing the WBC seems feasible and the pre/post results are encouraging as well.

The results showed that a rehabilitation program including WBC had an impact on cardiometabolic profile, physical performance, sleep quality and overall well-being, suggesting that it may be an effective adjunct therapy in the rehabilitation of post-COVID obese patients.

Interestingly, an increase in parasympathetic tone is suggested by the significant reduction in HR observed. Autonomic dysfunction is a major hypothesis for symptom persistence in long COVID, and WBC may have a role to play as an adjuvant therapy as it can act as a ‘training method’ for the autonomic nervous system [63,64]. Indeed, it is well established that WBC is effective in increasing post-exercise and resting heart rate variability (HRV), an indicator of increased parasympathetic tone activation [65,66,67].

To the best of our knowledge, this study includes the largest sample of PCC in patients with obesity undergoing multidisciplinary rehabilitation combined with WBC. 

Given its known rapid effects, WBC sessions started as early as a few days after admission and were performed early in the day with the aim of improving patients’ overall physical performance and increasing adherence and motivation to rehabilitation. 

In addition, no adverse events were observed in the sample patients, indicating that this type of treatment can be performed safely. 

These findings may be of particular interest in cases in which rehabilitation programs may be hindered by pain, inflammation, or fatigue, and highlight the importance of early rehabilitation support. It is important to note that our results could lead to the possible application of the WBC in the rehabilitation of other respiratory diseases with similar symptoms. In fact, in obstructive lung disease, the sympathetic nervous system may be affected by recurrent episodes of hypoxemia, hypercapnia, elevated intrathoracic airway pressures, increased ventilatory effort, systemic inflammation and beta-sympathomimetics [68]. Rehabilitation of these patients should consider treatments that aim to re-establish the sympathovagal balance to reduce resting sympathetic activity, such as WBC, exercise training, muscle stretching and breathing relaxation techniques [68,69].

Our data have some limitations, the main one being the lack of a control group of long-term COVID patients, which did not allow us to analyze both the evolution of the treated patients with the natural evolution of the symptoms, which is still unknown, and the effect of medical follow-up “alone”, in a dedicated facility and group. In the absence of a control group, our results do not fully clarify the extent to which WBC, the multidisciplinary rehabilitation program, or a combination of the two may account for the observed improvements. It is not always clear whether improvements in anthropometrics, blood tests and general well-being are due to exercise, diet, and psychological intervention alone, or to the addition of WBC.

Due to the wide range of PCC symptoms, the sample was heterogeneous and consisted of different degrees of obesity, associated physical abilities and comorbidities. 

Another limitation was the small number of participants involved in the study, therefore, larger studies with diverse populations should be conducted to determine the generalizability of these findings to a wider range of patients with PCC. 

The reported results may have been influenced by motivational factors related to participation in a novel, well-tolerated treatment. Despite these limitations, the results of this feasibility study provide valuable insights into the potential efficacy and impact of a multidisciplinary rehabilitation program incorporating WBC in PCC patients with obesity. Future research should aim to further investigate the long-term effects and benefits of WBC in the rehabilitation of PCC. Also, it would be valuable to investigate the most appropriate frequency, time, and temperature protocols. 

## 5. Conclusions

This study shows that a comprehensive multidisciplinary rehabilitation protocol that includes WBC developed for patients with PCC and obesity is safe and feasible. The overall improvement in physical performance, hematological and metabolic parameters, psychological and general well-being, and pain demonstrates that exercise rehabilitation was possibly an effective tool for long-term COVID patients. The clinical implications of this study are that WBC can be considered as an adjuvant and booster therapy in post-COVID rehabilitation of patients with obesity and PCC.

In addition, it is important to emphasize that the introduction of WBC was a turning point for all participants in terms of subjective and objective improvements in health and function, and that the overall improvement in clinical, physical, and biochemical parameters at discharge supports the use of WBC as an additional option in the multidisciplinary management of PCC. 

In conclusion, considering that the severity and prevalence of PCC in the general population is still high, the identification of rehabilitation programs and adjuvants that can act as a booster for rehabilitation programs appears to be of paramount importance. However, due to the heterogeneity of this condition, rehabilitation protocols should be tailored to each patient’s needs. 

The small sample size makes it difficult to draw conclusions but underlines the importance of establishing a rehabilitation pathway for the care of patients with long COVID that can be adapted and tailored to the individual symptoms. Larger randomized trials with diverse populations should be conducted to determine the generalizability of these findings to a wider range of post-COVID patients. 

## Figures and Tables

**Table 1:** Baseline clinical and demographic characteristics (N = 16).

	Mean	SD		Mean	SD
Age (y)	55.94	±7.50	CRP (mg/L)	0.57	±0.54
Weight (kg)	99.83	±29.19	HDL (mg/dL)	49.37	±11.41
BMI (kg/m^2^)	37.99	±8.44	LDL (mg/dL)	130.50	±40.94
WC (cm)	114.40	±13.99	Tot. Col. (mg/dL)	196.96	±49.06
LOS (d)	30.6	±7.69	TG (mg/dL)	156.62	±51.38
6MWT (m)	381.80	±145.05	Glu (mg/dL)	112.25	±40.70
TUG (s)	12.65	±11.65	SBP (mmHg)	135.0	±19.0
VAS pain	53.06	±26.64	DBP (mmHg)	81.3	±11.9
PGWBI	63.50	±16.92	HR (bpm)	80.0	±10.4

6MWT, 6-Minute Walk Test; BMI, Body Mass Index; bpm, beat per minute; CRP, C-Reactive Protein; DBP, Diastolic Blood Pressure; HDL, high-density lipoprotein; HR, Heart Rate; LDL, Low-Density Lipoprotein; LOS, Length of Stay; PGWBI, Psychological General Well-Being Index; SBP, Systolic Blood Pressure; SpO2, Saturation of Peripheral Oxygen; TG, triglycerides; Tot Col., Total Cholesterol; TUG, Timed Up and Go test; VAS, Visual Analogue Scale; WC, Waist Circumference.

**Table 2:** Comparison between the parameters at T0 and at T10 (N = 16).

	T0		T10		*p*-Value
Weight (kg)	99.83	±29.19	95.819	±27.10	**<0.001** ᵃ
BMI (kg/m^2^)	37.99	±8.44	36.491	±7.81	**<0.001** ᵇ
WC (cm)	114.40	±13.99	108.800	±13.73	**0.002** ᵃ
6MWT (m)	381.80	±145.05	446.200	±84.56	**0.093** ᵃ
TUG (s)	12.65	±11.65	9.373	±4.67	**0.009** ᵃ
VAS pain	53.06	±26.64	33.938	±18.71	**<0.001** ᵇ
PGWBI	63.50	±16.92	76.188	±16.59	**0.002** ᵃ
CRP (mg/L)	0.57	±0.54	0.459	±0.41	**0.013** ᵃ
HDL (mg/dL)	49.37	±11.41	43.688	±9.90	**<0.001** ᵇ
LDL (mg/dL)	130.50	±40.94	107.563	±47.00	**0.005** ᵇ
Tot. Col. (mg/dL)	196.96	±49.06	176.838	±56.51	**0.013** ᵇ
TG (mg/dL)	156.62	±51.38	147.438	±49.77	0.473 ᵇ
Glu (mg/dL)	112.25	±40.70	97.563	±17.94	**0.003** ᵃ
SBP (mmHg)	135.0	±19.0	125.625	±14.59	**0.046** ᵃ
DBP (mmHg)	81.3	±11.9	76.250	±5.00	0.084 ᵇ
HR (bpm)	80.0	±10.4	73.188	±5.50	**0.014** ᵇ
SpO_2_ *	91.5	±1.47	94.0	±2.05	**0.047** ᵇ

^a^, Wilcoxon test; ^b^, t-Student test. * Only patients admitted to Pneumology Unit. 6MWT, 6-Minute Walk Test; BMI, Body Mass Index; bpm, beat per minute; CRP, C-Reactive Protein; DBP, Diastolic Blood Pressure; HDL, high-density lipoprotein; HR, Heart Rate; LDL, Low-Density Lipoprotein; PGWBI, Psychological General Well-Being Index; SBP, Systolic Blood Pressure; SpO2, Saturation of Peripheral Oxygen; TG, triglycerides; Tot. Col., Total Cholesterol; TUG, Timed Up and Go test; VAS, Visual Analogue Scale; WC, Waist Circumference.

## Data Availability

Data are contained within the article.
